# Three-year clinical experience with magnetic sphincter augmentation and laparoscopic fundoplication

**DOI:** 10.1007/s00464-020-07792-1

**Published:** 2020-07-16

**Authors:** Luigi Bonavina, Thomas Horbach, Sebastian F. Schoppmann, Janet DeMarchi

**Affiliations:** 1 Division of General and Foregut Surgery, Department of Biomedical Sciences for Health, University of Milano, IRCCS Policlinico San Donato, Milano, Italy; 2 Department of Surgery, Schön Klinik Nürnberg Fürth, 90763 Fürth, Germany; 3 Department of Surgery, Medical University Vienna, 1090 Vienna, Austria; 4 Ethicon, Inc., Cincinnati, OH USA; 5 Ethicon, Inc., 15803 Sunset Rd, Minnetonka, MN 55345 USA

**Keywords:** Anti-reflux surgery, GERD, LINX, Fundoplication, Regurgitation, Proton pump inhibitors

## Abstract

**Background:**

Magnetic sphincter augmentation (MSA) is a surgical intervention for gastroesophageal reflux disease (GERD) which has been evaluated in numerous studies and has shown beneficial effects. Long-term effectiveness data for MSA as well as laparoscopic fundoplication (LF) in patients with GERD are needed.

**Objective:**

The objective of this study was to evaluate the 3-year outcomes for MSA and LF in patients with GERD.

**Methods:**

This prospective, multi-center, observational registry study evaluated MSA and LF in clinical practice over 3 years (ClinicalTrials.gov identifier: NCT01624506). Data collection included baseline characteristics, reflux symptoms, medication use, satisfaction and complications. Post-surgical evaluations were collected at yearly intervals.

**Results:**

Between December 2009 and December 2014, 631 patients (465 MSA and 166 LF) were enrolled in the registry. Both MSA and LF resulted in improvements in total GERD-HRQL score (mean reduction in GERD-HRQL from baseline to 3 years post-surgery: MSA 22.0 to 4.6 and LF 23.6 to 4.9) and in satisfaction (GERD-HRQL satisfaction increase from baseline to 3 years: MSA 4.6% to 78.2% and LF 3.7% to 76.5%). Most patients were able to belch as needed with both therapies (MSA 97.6% and LF 91.7% at 3 years). MSA allowed a higher percentage of patients the ability to vomit as needed (MSA 91.2% and LF 68.0% at 3 years). PPI usage declined from baseline to 3 years for both groups after surgery (MSA 97.8% to 24.2% and LF 95.8% to 19.5%). The mean procedure time was shorter for MSA than for LF. Intraoperative and procedure-related complication rates (≤ 2%) were low for both therapies.

**Conclusions:**

This 3-year prospective observational registry study contributes to the mounting evidence for the effectiveness of MSA and LF. Despite the more severe nature of GERD in the LF group, the clinical outcomes for MSA and LF were favorable from an effectiveness and safety standpoint.

Gastroesophageal reflux disease (GERD) is defined as symptoms or complications resulting from the reflux of gastric contents into the esophagus or beyond, into the oral cavity (including larynx) or lung 1]. Persistent and intense GERD symptoms have a significant and negative impact on both health-related quality of life (HRQL) and healthcare resource utilization [2]. Treatment options for people who suffer from GERD vary widely, depending on the severity and symptoms of their disease. There are currently three primary means of treating GERD: lifestyle changes, medical therapy, and surgical intervention [3]. Reasons to refer GERD patients for surgery may include: proton pump inhibitor (PPI) non-compliance, side effects associated with medical therapy, presence of a large hiatal hernia, esophagitis refractory to medical therapy, or persistent symptoms caused by refractory GERD [1, 4, 5].

The traditional surgical treatment for GERD is a laparoscopic fundoplication [6]. Nissen fundoplication involves wrapping a portion of the stomach around the entire esophagus(360°) to reinforce the weakened lower esophageal sphincter (LES). Another variation of fundoplication is the Toupet procedure which wraps a portion of the stomach around 270° of the esophagus. Yet a third variation is the Dor fundoplication which consists of wrapping a portion of the stomach around 180° of the esophagus. The fundoplication procedure has several shortcomings that have limited its use: (1) it results in anatomical and physiological alteration of the fundus; (2) potentially debilitating side effects including gas bloat and an inability to belch or vomit [7, 8]; (3) the procedure is not standardized, resulting in variable effectiveness [8]; and (4) it is a not a fully reversible procedure [9]. For the purpose of this publication, laparoscopic fundoplication (LF) includes Nissen, Toupet or Other/Unspecified fundoplication procedures. Magnetic sphincter augmentation (MSA) is an alternative to fundoplication which has been studied in diverse patient populations and has shown beneficial effects [5, 9–23]. MSA augments the weak lower esophageal sphincter (LES), restoring the body's natural barrier to reflux [24]. The LINX System is a small flexible band of interlinked titanium beads with magnetic cores. The magnetic attraction between the beads is intended to help the LES resist opening to gastric pressures, preventing reflux from the stomach into the esophagus.

The outcomes associated with laparoscopic fundoplication (LF) and MSA have previously been evaluated, including a prospective, multi-center 1-year real-world prospective registry evaluating MSA and LF by Reigler et al. [23]. The 1-year registry study findings showed that both MSA and LF resulted in significant improvements in reflux control, with similar safety and reoperation rates [23]. This same patient population was followed an additional 2 years to demonstrate the long-term safety and effectiveness outcomes of MSA and LF and is the basis of this publication.

## Methods

This was a prospective, multi-center, observational registry evaluating MSA and LF in clinical practice (ClinicalTrials.gov identifier: NCT01624506). The study was conducted under a common protocol and approved by each center’s Ethics Committee. Informed consent was obtained before enrolling patients into the study.

### Patient enrollment and study population

Twenty-two medical centers in four countries (Austria, Germany, Italy, and the United Kingdom) enrolled all patients who were candidates for a surgical anti-reflux procedure. All patients had a diagnosis of GERD confirmed by abnormal esophageal acid exposure on a prolonged pH or pH impedance study and chronic reflux symptoms despite the daily use of medical therapy with PPIs. Patients with severe GERD, defined as meeting one or more of the following conditions, were also included: large hiatal hernia (> 3 cm diameter of the esophageal hiatus), Barrett’s esophagus, motility disorder, and Grade C or D esophagitis by Los Angeles (LA) classification. Patients without advanced GERD characteristics were considered to have moderate GERD (abnormal esophageal pH, reflux symptoms despite medication). Patients were excluded if they had known conditions that would make it unlikely for them to complete the 3-year follow-up (e.g., life expectancy < 3 years).

### Study procedures and outcomes

The type of anti-reflux procedure performed (MSA or LF [Nissen, Toupet or Other/Unspecified]) was provisionally agreed upon by the surgeon in close consultation with the patient. If a patient met the labeling requirements for MSA (hiatal hernia ≤ 3 cm, esophagitis less than Grade C, absence of Barrett’s esophagus, absence of motility disorders), MSA was recommended. Motility disorders were defined as having distal esophageal pressure less than 35 mm/Hg (or HRM equivalent of a DCI < 450), less than 70% effective swallows, or known disorders such as achalasia, nutcracker esophagus, diffuse esophageal spasm or a hypertensive lower esophageal sphincter. The MSA device was placed utilizing the minimal dissection technique. For those patients with larger hiatal hernias, more severe esophagitis, Barrett’s esophagus or some types of dysmotility, LF was recommended. The final choice of procedure was made by the surgeon at the time of laparoscopy. A patient may have been a MSA candidate based upon the preoperative screening but upon entering the abdomen, a surgeon may have encountered a larger than anticipated hiatal hernia or was unable to obtain enough intra-abdominal esophageal length to safely implant the MSA device. In this instance, a fundoplication would be performed. The majority of the LF group underwent Nissen fundoplication (62%). A Toupet fundoplication was performed in 31% of patients in the LF group with the remaining 7% undergoing an Other/Unspecified procedure. Postoperative care was directed by the surgeon based on the patient’s clinical condition and practices of the institution.

Data collection included baseline characteristics and pre- and post-surgical HRQL (evaluated with Gastroesophageal Reflux Disease-Health-Related Quality of Life (GERD-HRQL) symptom severity instrument) [25], patient satisfaction (GERD-HRQL satisfaction with present condition [proportion of satisfied, neutral, or dissatisfied]), symptoms, and use of PPIs (proportion of patients regularly taking PPIs), (GERD-HRQL PPI medication status) as well as the duration of surgery, length of stay, complications, and healthcare resource use. The GERD-HRQL was developed to measure symptomatic outcomes and therapeutic effects in patients with GERD [26]. The scale has 11 items which focus on heartburn symptoms, dysphagia, medication effects, and the patient's present health condition. Each item is scored from 0 to 5, with a higher score indicating a better quality of life.

Study-related questionnaires were administered to all patients prior to and 1, 2, and 3 years after surgery. The hospital staff administering the standardized questionnaires were unaware of the procedure that was performed. Pre- and post-surgery questionnaires were completed at the appropriate time points (i.e., preoperatively, at time of surgery, from 6 weeks to 6 months, at 12 months, at 24 months, and at 36 months). All registry follow-up visits had a ± 60-day visit window and could be conducted over the telephone. Both incorporated the GERD-HRQL, satisfaction with current condition, other GERD-related symptoms, reason for choosing anti-reflux surgery and perceived benefits of anti-reflux surgery. Use of PPIs was tracked before and after surgery. In addition, healthcare utilization related to procedural complaints or complications was tracked after surgery. Patients were also asked about their willingness to undergo surgery again.

### Study analyses

The registry was not a clinical study and therefore it was not powered to evaluate a study hypothesis. Statistical analyses were primarily descriptive. Means and standard deviations (SD) and/or medians were used to describe continuous variables and frequency counts and/or percentages summarized categorical variables. Statistical tests were only conducted for evaluating differences in baseline characteristics and for evaluating within-group changes over time. Two-tailed unpaired Student’s t test or the Wilcoxon rank sum test were used to compare continuous baseline characteristic values for MSA and LF. Chi-square tests or the Mann–Whitney were used to compare categorical baseline characteristic values for MSA and LF. Differences were considered to be significant at the 0.05 level. Analyses were performed using SAS version 9.4.

## Results

### Patient baseline demographic and clinical characteristics

Between Dec 2009 and Dec 2014, 631 patients (465 MSA and 166 LF) had enrolled in the registry. Patient baseline demographic and clinical characteristics for MSA and LF are presented in Table 1.

**Table 1:** Patient baseline demographic and clinical characteristics for MSA and LF

Measure	MSA *N* = 465	LF *N* = 166	*p* value
Age, years (mean ± SD)	46.6 ± 13.6	56.3 ± 12.6	< 0.0001
Gender, % of patients			0.866
Male	63.7%	49.4%	
Female	36.3%	50.6%	
BMI (kg/m^2^) (mean ± SD)	25.7 ± 3.7	27.81 ± 4.0	< 0.0001
Years with GERD (mean ± SD)	9.0 ± 7.7	9.2 ± 8.6	0.7950
Years of PPI Use (mean ± SD)	6.1 ± 5.3	5.7 ± 6.0	0.5184
Esophagitis, % of patients			0.0130
None	53.0%	40.9%	
Grade A	31.7%	29.6%	
Grade B	13.5%	16.4%	
Grade C	1.1%	8.2%	
Grade D	0.7%	5.0%	
Barrett’s Esophagus, % of patients	1.7%	12.7%	< 0.0001
Hiatal Hernia Size, % of patients			< 0.0001
None	19.7%	7.5%	
1–3 cm	78.9%	44.4%	
> 3 cm	1.4%	48.1%	
Total % Time pH < 4 (mean ± SD)	12.2 ± 11.4	13.0 ± 14.7	0.5830
Moderate GERD, % of patients^a^	90.8	18.1	
Severe GERD, % of patients^b^	9.2	81.9	

*BMI* body mass index, *GERD* gastroesophageal reflux disease, *LF* laparoscopic fundoplication, *MSA* magnetic sphincter augmentation, *PPI* proton pump inhibitor

^a^Moderate GERD defined as hiatal hernia ≤ 3 cm, no Barrett’s esophagus, no motility disorder, and esophagitis ≤ Grade B by LA Classification

^b^Severe GERD defined as one or more of: hiatal hernia > 3 cm, Barrett’s esophagus, motility disorder and/or Grade C or D esophagitis by LA Classification

The particular patient baseline characteristics that were statistically significantly different between patients with MSA vs. LF (all *p* < 0.0001) were patient age (LF 56.3 years vs. MSA 46.6 years), BMI (LF 27.8 vs MSA 25.7), frequency of large hiatal hernias (LF 48.1% vs. MSA 1.4%), and the presence of Barrett’s esophagus at the time of surgery (LF 12.7% vs MSA 1.7%). Also, a greater proportion of patients with MSA had no esophagitis (*p* = 0.0130). It is notable that approximately 91% of patients in the MSA group met the definition of moderate GERD (hiatal hernia absent or ≤ 3 cm, normal motility, no Barrett’s esophagus and esophagitis ≤ Grade B by LA Classification), while just 18% of the LF group were categorized as having moderate GERD. On the other hand, approximately 83% of patients in the LF group met the criteria for severe GERD (one of more of the following: hiatal hernia > 3 cm, Barrett’s esophagus, motility disorder and/or esophagitis Grade C or D by LA Classification), while 9% of the MSA group met the criteria for severe GERD. Given this difference in severity between the two groups, the median GERD-HRQL scores were similar with 22.0 for the MSA group and 23.0 for the LF group but not statistically significant with a *p* value of 0.0620. Patients did not differ in other evaluated baseline characteristics.

### Clinical effectiveness and HRQL of MSA and LF over time

Table 2 presents the clinical effectiveness of MSA and LF pre- and post-surgery.

**Table 2:** Clinical effectiveness of MSA and LF pre- and post-surgery

Measure	MSA % (*n*/*N*) [95% CI]	LF % (*n*/*N*) [95% CI]
Satisfaction with current condition (from GERD-HRQL)		
Baseline	4.6% (21/460) [2.7%, 6.5%]	3.7% (6/164) [0.8%, 6.5%]
12 M	75.3% (326/433) [71.2%, 79.4%]	77.2% (122/158) [70.7%, 83.8%]
24 M	78.9% (254/322) [74.4%, 83.3%]	83.3% (90/108) [76.3%, 90.4%]
36 M	78.2% (230/294) [73.5%, 82.9%]	76.5% (65/85) [67.5%, 85.5%]
GERD interfering with sleep		
Baseline	73.3% (333/454) [69.3%, 77.4%]	78.0% (128/164) [71.7%, 84.4%]
12 M	11.9% (50/419) [8.8%, 15.0%]	9.6% (15/157) [5.0%, 14.2%]
24 M	11.7% (37/315) [8.2%, 15.3%]	5.5% (6/109) [1.2%, 9.8%]
36 M	9.0% (26/290) [5.7%, 12.3%]	10.7% (9/84) [4.1%, 17.3%]
Ability to belch		
Baseline	96.7% (441/456) [95.1%, 98.3%]	93.9% (154/164) [90.2%, 97.6%]
12 M	96.7% (406/420) [94.9%, 98.4%]	88.5% (138/156) [83.4%, 93.5%]
24 M	97.2% (308/317) [95.3%, 99.0%]	92.5% (99/107) [87.5%, 97.5%]
36 M	97.6% (284/291) [95.8%, 99.4%]	91.7% (77/84) [85.8%, 97.6%]
Ability to vomit^a^		
Baseline	96.6% (343/355) [94.8%, 98.4%]	92.0% (115/125) [87.2%, 96.8%]
12 M	89.7% (191/213) [85.6%, 93.8%]	55.8% (29/52) [42.3%, 69.3%]
24 M	85.8% (133/155) [80.3%, 91.3%]	52.6% (20/38) [36.7%, 68.5%]
36 M	91.2% (134/147) [86.6%, 95.8%]	68.0% (17/25) [49.8%, 86.2%]
Use of PPIs		
Baseline	97.8% (453/463) [95.6%,100%]	95.8% (158/165) [91.6%, 100%]
12 M	18.9% (81/428) [15.2%, 22.6%]	19.7% (31/157) [13.5%, 26.0%]
24 M	21.4% (74/346) [17.1%, 25.7%]	18.1% (21/116) [11.1%, 25.1%]
36 M	24.2% (76/314) [19.5%, 28.9%]	19.5% (17/87) [11.2%, 27.9%]
Willingness to have surgery again		
12 M	89.5% (366/409) [86.5%, 92.5%]	91.1% (143/157) [86.6%, 95.5%]
24 M	90.6% (281/310) [87.4%, 93.9%]	94.4% (102/108) [90.1%, 98.8%]
36 M	93.1% (270/290) [90.2%, 96.0%]	94.0% (79/84) [89.0%, 99.1%]

*CI* confidence interval, *GERD* gastroesophageal reflux disease, *HRQL* health-related quality of life, *LF* laparoscopic fundoplication, *M* months, *MSA* magnetic sphincter augmentation, *PPI* proton pump inhibitor

^a^Data adjusted to include only those patients who found the need to vomit

Both MSA and LF resulted in an improvement in satisfaction (satisfaction score from GERD-HRQL) over the 1, 2, and 3 years after surgery. The proportion of patients whose sleep was affected by GERD declined upon surgery for both MSA and LF. At 24 months, the proportion of MSA patients with sleep problems was twice that of LF patients; however, the confidence intervals overlapped and these differences were not observed at 36 months. Despite the one-way valve of LF and the two-way physiologic valve of MSA, patients appear to be able to belch as needed with both therapies. It is notable that at each postoperative time point, MSA allowed a higher percentage of patients the ability to vomit if needed with 91.2% of patients noting the ability to vomit at 36 months. At the same timepoint, 68% of the LF patients were able to vomit if needed. PPI usage also declined for both groups after surgery. Most patients reported a willingness to have the surgery again throughout the 3-year follow-up period.

Both MSA and LF resulted in substantial improvements in total GERD-HRQL score (a lower GERD-HRQL score signifies higher quality of life) from baseline over the 1, 2, and 3 years after surgery. This reduction, seen in both groups, was statistically significant and maintained over the entire study period which shows appreciable durability of the therapeutic effect of both anti-reflux surgical options. The results of this analysis are summarized in Table 3.

**Table 3:** GERD-HRQL scores and change from baseline

Measure	MSA		LF	
	Mean GERD-HRQL ± SD Median (Min, Max)	Mean Change from Baseline ± SD Median (Min, Max) [95% CL] *p* value	Mean GERD-HRQL ± SD Median (Min, Max)	Mean Change from Baseline ± SD Median (Min, Max) [95% CL] *p* value
Baseline	*n* = 457 22.0 ± 9.1 22.0 (0.0, 47.0)		*n* = 163 23.6 ± 9.8 23.0 (3.0, 47.0)	
Paired Baseline/Month 12	*n* = 414 21.9 ± 9.0 22.5 (0.0, 46.0)		*n* = 152 23.4 ± 9.9 23.0 (3.0, 47.0)	
Month 12	*n* = 418 5.2 ± 6.4 3.0 (0.0, 42.0)	*n* = 414 − 16.7 ± 10.0 − 17.0 (− 41.0, 21.0) [− 17.6, − 15.7] < 0.001	*n* = 154 4.9 ± 7.2 3.0 (0.0, 48.0)	*n* = 152 − 18.5 ± 11.5 − 19.5 (− 45.0, 20.0) [− 20.3, − 16.6] < 0.001
Paired Baseline/Month 24	*n* = 296 21.6 ± 9.2 22.0 (0.0, 41.0)		*n* = 103 23.9 ± 10.1 24.0 (3.0, 47.0)	
Month 24	*n* = 300 4.9 ± 6.1 2.0 (0.0, 35.0)	*n* = 296 − 16.7 ± 10.6 − 17.0 (− 39.0, 28.0) [− 17.9, − 15.5] < 0.001	*n* = 105 3.9 ± 4.4 3.0 (0.0, 19.0)	*n* = 103 − 20.0 ± 10.0 − 20.0 (− 45.0, 0.0) [− 22.0, − 18.1] < 0.001
Paired Baseline/Month 36	*n* = 278 21.3 ± 9.3 22.0 (0.0, 41.0)		*n* = 80 22.5 ± 9.7 22.5 (3.0, 47.0)	
Month 36	*n* = 283 4.6 ± 6.0 3.0 (0.0, 39.0)	*n* = 278 − 16.6 ± 10.2 − 18.0 (− 41.0, 12.0) [− 17.8, − 15.4] < 0.001	*n* = 82 4.9 ± 7.1 3.0 (0.0, 45.0)	*n* = 80 − 17.8 ± 10.6 − 18.0 (− 39.0, 17.0) [− 20.1, − 15.4] < 0.001

*CL* confidence limit, *GERD* gastroesophageal reflux disease, *HRQL* health-related quality of life, *LF* laparoscopic fundoplication, *max* maximum, *min* minimum, *MSA* magnetic sphincter augmentation, *SD* standard deviation

With respect to preoperative and postoperative dysphagia, both MSA and LF groups reported daily, bothersome dysphagia at baseline with 15.7% and 24.4%, respectively. Both groups showed a decrease postoperatively, summarized in Table 4.

**Table 4:** Dysphagia results for MSA and Fundoplication over study duration

Timepoint	MSA	Fundo	Q7 *p* value
Baseline			
Score	1.0 ± 1.3	1.3 ± 1.5	0.0227
% Q7 > 3.0	15.7%	24.4%	0.0174
12 months			
Score	0.8 ± 1.1	0.6 ± 1.1	–
% Q7 > 3.0	8.8%	7.6%	
24 months			
Score	0.6 ± 0.9	0.4 ± 0.9	–
% Q7 > 3.0	4.4%	4.6%	
36 months			
Score	0.5 ± 0.9	0.4 ± 1.1	–
% Q7 > 3.0	3.8%	4.8%	

When looking at the LF group and dysphagia, the percentage of patients reporting daily bothersome dysphagia was similar across the three procedure options, as outlined in Table 5.

**Table 5:** Dysphagia results for Nissen, Toupet and Other over study duration

Timepoint	Nissen	Toupet	Other
Baseline			
Score	1.3 ± 1.6	1.3 ± 1.4	1.7 ± 1.5
% Q7 > 3.0	26.7% (27/101)	19.6% (10/51)	25.0% (3/12)
12 months			
Score	0.7 ± 1.1	0.6 ± 1.1	0.8 ± 1.3
% Q7 > 3.0	7.4% (7/95)	8.0% (4/50)	8.3% (1/12)
24 months			
Score	0.5 ± 0.9	0.4 ± 0.9	0.5 ± 0.9
% Q7 > 3.0	4.4% (3/68)	6.3% (2/32)	0.0% (0/8)
36 months			
Score	0.4 ± 1.0	0.5 ± 1.1	0.4 ± 0.9
% Q7 > 3.0	5.9% (3/51)	3.7% (1/27)	0.0% (0/5)

### Procedure time, duration of hospital stay, and clinical safety of MSA and LF

The procedure time, duration of hospital stay, and clinical safety of MSA and LF are presented in Table 6.

**Table 6:** Procedure time, duration of hospital stay, and clinical safety of MSA and LF

Measure	MSA (*n* = 459)	LF (*n* = 163)
Mean procedure time (min)	43.2 ± 19.7	79.7 ± 47.7
Length of Stay < 24 h	36.1%	11.4%
Length of Stay > 48 h^a^	50.8%	72.3%
Intraoperative complication rate	1.8%	1.2%
Procedure-related complication rate	2.0%	1.8%

*LF* laparoscopic fundoplication, *MSA* magnetic sphincter augmentation

^a^238 of 465 were German patients, have longer stay built into reimbursement

The mean procedure time was 43.2 min for MSA compared with 79.7 min with LF. More than one-third of patients with MSA had a length of stay < 24 h versus 11.4% of patients with LF. Nearly three-quarters of patients with LF had a length of stay > 48 h versus 50.8% of patients with MSA. The intraoperative and procedure-related complication rates were low (≤ 2%) and similar for MSA and LF.

### Healthcare resource use with MSA and LF

The healthcare resource use for patients treated with MSA and LF is presented in Table 7.

**Table 7:** Healthcare resource use with MSA and LF

Measure	MSA (*n* = 459)	LF (*n* = 163)
Outpatient clinic visits		
12 M	18.9%	15.3%
24 M	14.7%	12.9%
36 M	10.5%	8.0%
Return to clinic for GERD symptoms		
12 M	58.5%	54.2%
24 M	80.4%	86.7%
36 M	87.9%	100%
Return to clinic due to procedural complaint/complication		
12 M	39.3%	41.7%
24 M	19.2%	20.0%
36 M	15.2%	0.0%
Surgical intervention		
12 M	1.6%	1.9%
24 M	1.2%	0.0%
36 M	0.6%	0.0%
Device removal*		
12 M	1.5% (7/459)	NA
24 M	2.0% (9/459)	NA
36 M	2.4% (11/459)	NA

*GERD* gastroesophageal reflux disease, *LF* laparoscopic fundoplication, *MSA* magnetic sphincter augmentation

*Removal rates are cumulative across the 3 years

The proportion of patients with outpatient clinic visits for GERD symptoms or due to procedural complaints or complications was similar for MSA and LF over the 3 years. Most outpatient clinic visits were due to GERD symptoms. In the early postoperative period, approximately 40% of outpatient visits in both MSA and LF groups were for procedure complaints; however, this rate trended downwards as expected over time. The surgical intervention rates and the device removal rate remained low over the 3 years. The surgical intervention rate for the MSA group at 3 years was 2.4% (11/459). The LF group had a surgical intervention rate of 1.9% (3/157). The intervention for the MSA group was the removal of the device for dysphagia (45%), ongoing GERD (18%), vomiting/regurgitation (18%), gastric pain (9.5%) and need for MRI (9.5%). There were no complications noted during the removal procedures. Two patients underwent fundoplication at the time of the device removal. The interventions for the LF group were revision of a Nissen wrap due to ongoing GERD, re-herniation and a sigmoid resection secondary to diverticulitis. No complications or ongoing sequelae were reported.

## Discussion

This is the first long-term prospective study that looks specifically at the safety and effectiveness outcomes of MSA and LF. This 3-year prospective, observational registry study contributes to the mounting real-world evidence for the effectiveness of MSA and LF. Randomized controlled trials (RCTs) remain the gold standard for assessing efficacy; however, they are inadequate for addressing questions about real-world effectiveness and safety of interventions [27, 28]. The high degree of internal validity with RCTs comes at the price of reduced external validity because study populations, protocols, and circumstances may not be relevant to the 'real-world' or diverse populations [29]. Specific to the GERD treatment options of a type of fundoplication and magnetic sphincter augmentation, randomization may not be appropriate due to the inherent variability in the fundoplication procedure as opposed to the standardized magnetic sphincter augmentation procedure. Patients who have researched the options often request one treatment over the other and would not accept leaving the treatment option to chance. There is also an inherent bias built into the patient selection for the two treatments. This study concluded that the patients who received MSA were less severe by definition. The Instructions for Use (IFU) for MSA and therefore inclusion criteria at the time of this trial, excluded hiatal hernias > 3 cm, Barrett’s esophagus, motility disorders and more significant esophagitis. These are common findings in the population affected by GERD. The surgeon, in following the IFU for MSA, would generally choose the less severe patient who would fit the criteria. It would be difficult to randomize this group because there are patients who would not be appropriate for MSA as outlined in the current labeling, such as those with large hiatal hernias, Barrett’s esophagus or motility disorders, who may benefit from a type of fundoplication instead. Even so, given these challenges, a randomized controlled study would contribute to the evidence base for these two surgical procedures. Unfortunately, such a study was proposed to the United Kingdom’s Medical Research Council in 2012 but the funding was denied [30]. Comparative effectiveness research (CER) focuses on real-world evidence to assist consumers, clinicians, purchasers, and policymakers to make informed decisions that will improve healthcare at the individual and population levels [31]. Real-world observational studies can address many of the limitations of RCTs because they leverage data originating from clinical practice and better reflect real-world conditions, a central concept of Comparative Effectiveness Research [32].

The findings from this study showed that the clinical outcomes for MSA and LF were favorable in regards to effectiveness, quality of life, satisfaction, PPI use, and safety, with low rates of complications and similar rates of clinic visits over 3 years. These results are in alignment with previously published effectiveness data for MSA and LF which showed that MSA and LF both lead to a decrease in HRQL score and an increase in patient satisfaction when compared with patient's preoperative symptoms [33, 34]. The proportion of patients with the ability to belch with LF in our study increased over the first 2 years and then stayed relatively steady, possibly due to the loosening of the fundoplication over time (ie, transthoracic migration of the wrap) [35, 36]. At 3 years, in the MSA cohort, 91.2% of patients reported the ability to vomit with just 68.0% of the LF group having the ability to do so.

The previously published 1-year registry results by Riegler et al., [23] which used unadjusted data, showed that the median GERD-HRQL score improved from 20.0 to 3.0 after MSA and 23.0 to 3.5 after LF. Moderate or severe regurgitation improved from 58.2% to 3.1% after MSA and 60.0% to 13.0% after LF (*p* = 0.014). Discontinuation of PPIs was achieved by 81.8% of patients after MSA and 63.0% after LF (*p* = 0.009). Excessive gas and abdominal bloating were reported by 10.0% of patients after MSA and 31.9% following LF (*p* < 0.001). Following MSA, 91.3% of patients were able to vomit if needed, compared with 44.4% of those undergoing LF (*p* < 0.001).

Aiolfi and colleagues performed a systematic review and meta-analysis of patients undergoing MSA or LF (Nissen or Toupet) [19]. Seven studies, published between 2014 and 2017, met the criteria for the analysis, providing a total of 1211 patients. The MSA cohort consisted of 686 patients (56%) and the LF cohort, those undergoing either laparoscopic full (Nissen) or partial (Toupet) fundoplication, included 525 patients (44%). The postoperative follow-up ranged from six to twelve months. In the entire cohort, the mean age ranged from 39.3 to 54 years. The mean hernia size ranged from 1 to 2 cm, esophagitis ≥ Grade B was present in 15.4%. Barrett’s esophagus was diagnosed in 16.2% of the patients. At up to 1 year, improvement in the quality of life scores, assessed by GERD-HRQL and the rate of PPI suspension were similar between the two groups. There were 13 reoperations in the MSA group (1.9%), consisting of 12 device removals and one crural release. In the LF group, there were 11 reoperations (2.1%) including herniation of the fundic wrap (5), persistent GERD (3), retro-esophageal abscess (2), crural release (1). The authors concluded that patients with GERD may benefit from both LF and MSA in terms of safety, postoperative quality of life, and PPI suspension at 1-year follow-up.

These findings are also consistent with long-term single-arm data for MSA. Saino et al. [13] conducted a prospective multi-center study at four clinical sites in the US and Europe and found that mean total GERD-HRQL score improved significantly from 25.7 to 2.9 (*p* < 0.001) with MSA when comparing baseline and 5 years (*n* = 33), and that 93.9% of patients had at least a 50% reduction in GERD-HRQL total score compared with baseline. Complete discontinuation of PPIs was achieved by 87.8% of patients. No complications occurred in the long-term, including no device erosions or migrations at any point. Similarly, Ganz and colleagues [37] conducted a prospective study of MSA in 100 adults who were partially responsive to daily PPIs at 14 centers in the US and The Netherlands. Median baseline GERD-HRQL scores were 27 in patients not taking PPIs and 11 in patients on PPIs; 5 years after MSA GERD-HRQL scores decreased to 4. All patients used PPIs at baseline; however, only 15.3% used them at 5 years. Moderate or severe regurgitation occurred in 57% of subjects at baseline, but only 1.2% at 5 years. All patients reported the ability to belch and vomit if needed. Bothersome dysphagia was present in 5% at baseline and in 6% at 5 years. Bothersome gas bloat was present in 52% at baseline and decreased to 8.3% at 5 years. A recent study by Ayazi et al. [38] on 553 patients at a single institution, looked specifically at identifying patient characteristics that may predict a positive outcome with MSA. Four of the independent predictors identified are age less than 45 years, male gender, GERD-HRQL preoperative score greater than 15 and an abnormal DeMeester score. These predictors align more closely with the MSA cohort in this study.

There are limitations associated with this analysis. MSA and LF effectiveness estimates were derived from 22 medical centers in four European countries and are not necessarily representative of all settings of care. All investigators performed fundoplication routinely and in addition, were trained specifically on the implantation of MSA which is only available in select centers. Within the LF group, there were different procedures performed at the discretion of the surgeon. While this individualizes the treatment to the patient, it makes the analysis more complex. This was a non-randomized study and was not intended to detect statistically significant clinical outcomes between MSA and LF. This registry was intended to capture everyday clinical practice and patients were screened based upon the current Instructions for Use (IFU) for MSA as those who met the MSA criteria. The final decision for the type of surgery was made by the surgeon at the time of laparoscopy, based upon a variety of patient-specific factors. Comparisons of baseline characteristics indicated that patients in the LF group were older, had a higher BMI, and had a greater frequency of large hiatal hernias and Barrett’s esophagus. It is possible that results were confounded and observed differences between groups were due to these and other variables. Due to the noted differences in the two populations, the results must be carefully considered. Furthermore, the procedure to implant MSA has since evolved to include full crural and gastroesophageal junction dissection as opposed to the minimal dissection utilized in this investigation. The timeframe for this study will determine if the procedural modifications are relevant to the outcomes in this study population. The current procedure theoretically may provide better outcomes for patients as compared to those done under the “minimal dissection” protocol as the hiatal hernia is often addressed.

An unmet need exists for the effective management of some refractory and/or chronic patients with GERD. In the current climate of tighter budgets and stricter management guidelines, it is important for health systems to assess both the clinical and economic value of surgical interventions and technologies. While they constitute a small proportion of the overall GERD population, GERD patients who are eligible for surgery present a significant clinical and socioeconomic burden. It is important to note that patients in both groups, on average, had suffered with GERD for 9 years and had been medicating with PPIs for 6 years. Persistent reflux symptoms, despite PPI therapy, can cause mental health disorders, sleep disorders, and psychological distress to patients [9, 39, 40]. Persistent and intense GERD symptoms have a negative impact on HRQL and healthcare resource utilization, including lower work productivity, greater activity impairment, more hours missed from work due to health problems, more visits to both primary-care physicians and specialists, and more emergency room visits [40].

## Conclusions

This 3-year prospective, observational registry study contributes to the mounting evidence for the effectiveness of MSA and LF. Despite the more severe nature of GERD in the LF group, the clinical outcomes for both MSA and LF were favorable from an effectiveness (GERD-HRQL), satisfaction, PPI use, and safety standpoint, with similar rates of complications and clinic visits. The use of MSA or LF is associated with improved patient clinical and QOL outcomes and lower overall burden of GERD disease. The analyses demonstrated that the evidence supports the use of MSA and LF as effective treatment strategies for patients with GERD eligible for surgery.
